# Macropinocytosis of Extracellular Glutathione Ameliorates Tumor Necrosis Factor α Release in Activated Macrophages

**DOI:** 10.1371/journal.pone.0025704

**Published:** 2011-10-03

**Authors:** Neal S. Gould, Elysia Min, Brian J. Day

**Affiliations:** 1 Department of Medicine, National Jewish Health, Denver, Colorado, United States of America; 2 Department of Pharmaceutical Sciences, University of Colorado Denver, Aurora, Colorado, United States of America; 3 Department of Medicine, University of Colorado Denver, Aurora, Colorado, United States of America; 4 Department of Immunology, University of Colorado Denver, Aurora, Colorado, United States of America; Institut National de la Santé et de la Recherche Médicale (INSERM), France

## Abstract

A number of inflammatory lung diseases have abnormally low glutathione (GSH) levels in the airway fluids. Lung macrophages are common mediators of inflammation, make up the majority of cells that are found in the airway epithelial lining fluid (ELF), and are commonly elevated in many lung diseases. Several animal models with altered ELF GSH levels are associated with similar alterations in the intracellular GSH levels of bronchoalveolar lavage (BAL) cells. The possible mechanisms and outcomes for this association between ELF GSH levels and intracellular BAL cell GSH are unknown. To investigate these issues, macrophages were grown in media supplemented with 500 µM GSH. GSH supplementation resulted in a 2–3 fold increase in macrophage intracellular GSH levels. The increase in macrophage intracellular GSH levels was associated with a significant reduction in NF-κB nuclear translocation and tumor necrosis factor α (TNFα) release upon LPS stimulation. Furthermore, co-treatment of macrophages with GSH and inhibitors of GSH breakdown or synthesis did not block GSH accumulation. In contrast, treatment with cytochalasin D, an inhibitor of actin dependent endocytosis, and amiloride, an inhibitor of macropinocytosis blocked, at least in part, GSH uptake. Furthermore, using two cigarette smoke exposure paradigms that result in two different GSH levels in the ELF and thus in the BAL cells resulted in modulation of cytokine release when stimulated with LPS ex vivo. These data suggest that macrophages are able to utilize extracellular GSH which can then modulate inflammatory signaling in response to proinflammatory stimuli. This data also suggests the lung can modulate inflammatory responses triggered by proinflammatory stimuli by altering ELF GSH levels and may help explain the dysregulated inflammation associated with lung diseases that have low ELF GSH levels.

## Introduction

The epithelial lining fluid (ELF) of the lung is a heterogeneous mixture of cells, proteins, and low molecular weight antioxidants [1], [2]. The ELF functions as a barrier and sensor for inhaled agents and pathogens [3]. The lung has developed adaptive mechanisms in which antioxidants, of which glutathione (GSH) is highly abundant, can be raised in the ELF in response to stressors [4], [5]. Additionally there are leukocytes that reside in the ELF and function to clear debris or pathogens that may deposit in the airways. Alveolar macrophages (AMs) make up between 88–95% of all the types of leukocytes typically recovered in bronchoalveolar lavage fluid (BALF) under normal conditions [6].

There are several lung diseases that have been shown to have characteristically low ELF GSH levels. These lung disorders include chronic obstructive pulmonary disease (COPD), acute respiratory distress syndrome (ARDS), cystic fibrosis (CF), and although not typically thought of as a disease, aging [7], [8], [9]. The decrease in ELF GSH (up to 90%) leaves these individuals incredibly susceptible to oxidant or pathogen mediated lung damage. Under conditions of decreased GSH, patients typically exhibit decreased pathogen clearance leading to chronic inflammation [10]. This is especially important since many of these lung disease have high levels of airway inflammation and recurrent exacerbations [11].

Exaggerated airway inflammation is a hallmark of COPD [12]. In models of COPD, the proinflammatory cytokine tumor necrosis factor alpha (TNFα) has been shown to be responsible for roughly 70% of the morphological changes associated with smoking, a major risk factor for COPD [13]. Additionally, in models of aging, ELF GSH levels have been shown to be inversely correlated with TNFα levels [14]. Furthermore, GSH has been directly linked with TNFα through the depletion of GSH with buthionine sulfoximine (BSO) resulting in the increase in airway TNFα levels [14].

One potential consequence of changes in ELF GSH may involve the AMs that reside in the ELF. When activated by a stimulus like cigarette smoke or lipopolysaccharide (LPS), macrophages produce and release TNFα and AMs have been shown to be highly activated when ELF GSH levels are low [15], [16]. However the mechanisms by which alveolar macrophages sense and respond to changes in ELF GSH are unknown.

In the present study macrophages were supplemented with extracellular GSH at physiologically relevant levels seen in the ELF and GSH synthesis dependant and independent pathways were examined. Additionally, the effect of altering GSH levels on macrophage TNFα release was assessed. These studies suggest that macrophages can uptake extracellular GSH by endocytosis and thereby alter their intracellular GSH levels resulting in suppressed cytokine response to inflammatory stimuli. These studies suggest a physiological role for maintaining high levels of ELF GSH in response to inflammatory stimuli as well as suggest a mechanism for the exaggerated inflammation seen in the number of lung disease states with low ELF GSH.

## Methods

### Animals and cells lines

C57B/6 mice were obtained from Jackson laboratory and aged mice were either obtained from our in house animal colony or the National Institute on Aging. Male 2 to 4 month old CFTR transgenic mice that posses the S480X truncation mutation with gut corrected recombinant human CFTR were obtained from our in house colony as previously reported [17]. All animal procedures were approved by the National Jewish Health IACUC committee. The murine macrophage like J774 cell line was obtained from the American Type Culture Collection and maintained in DMEM with 10% FBS and antibiotics.

### Bronchoalveolar lavage

Bronchoalveolar lavage (BAL) was performed using two 750 µL rinses of cold isotonic potassium phosphate solution. BAL cells were removed by centrifugation and analyzed for GSH. The dilution of the ELF was calculated by measuring urea in both the BALF and plasma as previously reported [14].

### Cigarette smoke exposure and L-Buthionine-sulfoximine (BSO) treatment

The mice were exposed to cigarette smoke (CS) from Kentucky reference cigarette 3R4F (University of Kentucky) for 2 h and sacrificed immediately following the exposure or for 24 h which includes 5 h CS exposure and 19 h rest period using a TE-10 cigarette smoke exposure system (Teague Enterprises). The average particulate matter was 120 mg/m^3^ and carbon monoxide averaged 350 ppm. For the pharmacologic manipulation of ELF GSH levels mice were administered BSO (Sigma) in the drinking water at a final concentration of 20 mM for a total of 11 days as previously reported [14].

### Primary alveolar macrophage (AM) isolation

Primary AM were isolated and pooled from the airways of 5–10 mice. Mice were given an overdose of pentobarbital and their tracheas were cannulated. BAL was performed four times using 1 mL of room temperature PBS. The recovered BALF was pooled and the centrifuged to pellet the lavage cells. The BAL cell pellet was resuspended in warm DMEM with 10% FBS and antibiotics and cultured in 96-well plates at a density of at least 70,000 cells per well. An aliquot of BAL cells was used to prepare cytospin slides for differential cell counts. Over 95% of the BAL cells recovered were AMs.

### Measurement of glutathione

Total glutathione (GSH) was measured spectrophotometrically as previously reported [14]. In short, 20 µL sample or standard in triplicate was incubated with 100 µL 5,5′-dithiobis(2-nitrobenzenoic acid) and glutathione reductase solution. The reaction was started by the addition of 50 µL NADPH and read at 412 nm for 5 min using a SpecraMax 340PC microplate reader (Molecular Devices). Intracellular levels are normalized to protein content.

### Tumor Necrosis Factor-α release

Pseudomonas LPS at a final concentration of 10 or 50 ng/mL was used to stimulate macrophage TNFα release. Tumor necrosis factor alpha (TNFα), a common proinflammatory cytokine, was measured in the cell culture media after 2 h stimulation with lipopolysaccharide (LPS). TNFα was measured by ELISA (BD Biosciences) according to manufactures recommendations.

### Glutathione supplementation

Cell culture media was supplemented with reduced glutathione at a final concentration of 500 µM. For the cell line, GSH was added to the media for 24 hours and only 2 hours for the primary AM before the assessment of changes in intracellular GSH levels. A stock fluorescent labeled GSH (Fl-GSH) solution was prepared by incubating GSH with monobromobimane at a 4∶1 ratio and then diluting to the desired concentration in the culture media, which should result in only a fraction of the total GSH that was added to the media as being fluorescent. For the in vivo administration of Fl-GSH, a 4 mM stock concentration of GSH was incubated with 3 mM monobromobimane and 20 µL was intra-tracheal administered for 2 h. Fluorescently labeled GSH was visualized in BAL cell cytospin preparations using an inverted EvoS fluorescent microscope (Advanced Microscopy Group) and quantified in the ELF and recovered macrophages using HPLC with fluorescent detection as previously described [17].

### Glutathione attenuation of TNFα release

Cells were cultured in media with or without GSH supplementation for 24 h to raise intracellular GSH levels. Old media was removed and fresh media was added with or without GSH and LPS at 10 or 50 ng/mL for a total of 2 h. Media was then removed and any non adherent cells were removed by centrifugation. TNFα in the media was then measured by ELISA. For primary AM, cells were freshly isolated and plated in media with or without GSH. LPS (10 or 50 ng/mL) was added immediately and the media was then removed after 2 h and TNFα analyzed by ELISA.

### Examination of GSH uptake pathway

To elucidate the uptake mechanism of GSH, inhibitors of several pathways were used. Buthionine-sulfoximine (BSO), an inhibitor of GSH synthesis, and acivicin, an inhibitor of GSH breakdown, were used at a final concentration of 100 µM concurrently with GSH in the media. Cytochalasin D, an inhibitor of endocytosis through the inhibition of actin polymerization, was used at a final concentration of 1 µM. Additionally more specific inhibitors such as amiloride (125 µM) for macropinocytosis, and polyinositol acid and amantadine (100 µM each) for receptor mediated pathways were also examined.

### Western blotting for protein expression

Nuclear and cytosolic fractions were obtained using the NE-PER nuclear isolation kit (Thermo) according to manufactures protocol. Total protein (10 µg) was loaded and run on a polyacrylamide gel and transferred to PVDF membrane. Membranes were blocked with 5% BSA and probed with primary antibodies for NF-κB p65 subunit at 1∶500 (Abcam) for 2.5 h at room temperature. The blots were then washed three times with tris buffered saline containing 1% tween 20 (TBS-T) and probed with the secondary peroxidase conjugated goat anti rabbit antibody (Abcam) at a dilution of 1∶25,000 for 30 min at room temperature. Proteins were visualized using ECL plus western blotting detection reagents (GE healthcare) according to manufactures protocol.

### Statistical analysis

All data is represented as the mean ± standard error of the mean. Significance was set at a p<0.05. An unpaired t test or two-way analysis of variance (ANOVA) was performed with a bonferroni post test using Prism 5 software (GraphPad).

## Results

### A correlation between bronchoalveolar lavage cell GSH levels and ELF GSH levels

The bronchoalveolar lavage (BAL) cells, which are predominately AMs, reside within the ELF, and thus many of the nutrients for them to survive are derived from the ELF. GSH is one of the most abundant antioxidants in the ELF and we hypothesized that the AMs may be able to utilize this large pool of GSH. In mouse models that have either normal, increased, or decreased ELF GSH we observed similar changes in the intracellular GSH levels from the recovered AM (Table 1). There is a strong positive correlation between AM GSH and ELF GSH levels (r = 0.72, p<0.0001). Under normal conditions the ELF GSH is roughly 96 µM and the AM GSH is 6.3 nmol/mg. When the ELF GSH increases to 272 µM in response to CS the AM GSH increased to 9.9 nmol/mg. Consequently, when very acute cigarette smoke exposures are performed, with little rest time between the exposure and sampling of the ELF, GSH levels are decreased to roughly 21 µM and 1.22 nmol/mg for the ELF and BAL cells respectively. Furthermore, in other animal models when the ELF GSH falls by roughly half either due to aging, alterations in transport through mutant CFTR, or chronic BSO treatment, the AM GSH falls to roughly half of the normal level as well.

**Table 1:** Correlation between mouse ELF GSH and Alveolar Macrophage GSH.*

	*ELF GSH (µM)*	*Alveolar Macrophage GSH (nmol/mg)*
Wild Type	96.6±1.57	6.3±0.75
2 h Cigarette smoke	21.52±8.11	1.22±0.32
24 h Cigarette smoke	615.4±82.47	11.54±0.42
recovery		
Aging (26 months)	46.6±3.96	3.3±0.84
CFTR KO	52.0±5.77	2.5±1.04
Chronic BSO	49.5±5.73	2.9±0.23

*Data represented as mean ±standard error, r = 0.72, p<0.0001.

### Macrophage utilization of extracellular GSH

**Figure 1:**
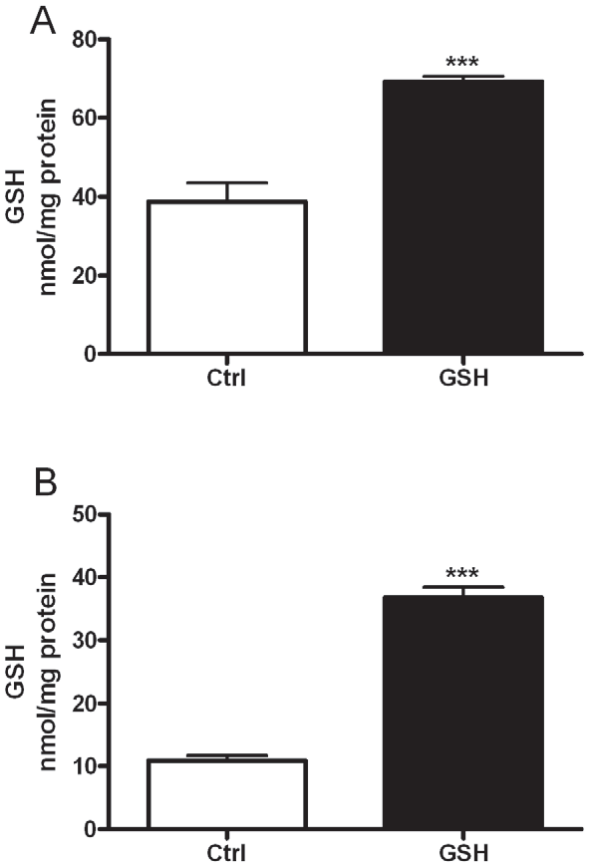
Extracellular GSH alters intracellular levels. J774 (A), or primary alveolar macrophages (B) were grown in media supplemented with or without 500 µM GSH and intracellular GSH levels were measured. Data represented as mean ± SEM, with ***p<0.001.

Based on the fact that there is a significant correlation between ELF GSH and macrophage GSH, we sought to examine whether macrophages can actually utilize extracellular GSH. Two different cell types, a murine derived macrophage cell line J774 (Fig. 1A), and primary mouse AM (Fig. 1B) were grown either in normal media or media supplemented with 500 µM GSH. 500 µM GSH was used because it is a physiologically relevant level that can easily be reached in the ELF in various times of stress including smoking [14] and infection [18]. When both the J774 and primary AM are grown in high GSH media the intracellular GSH levels were significantly higher than in cells grown in normal media.

### Intracellular GSH status alters inflammatory cytokine release

**Figure 2:**
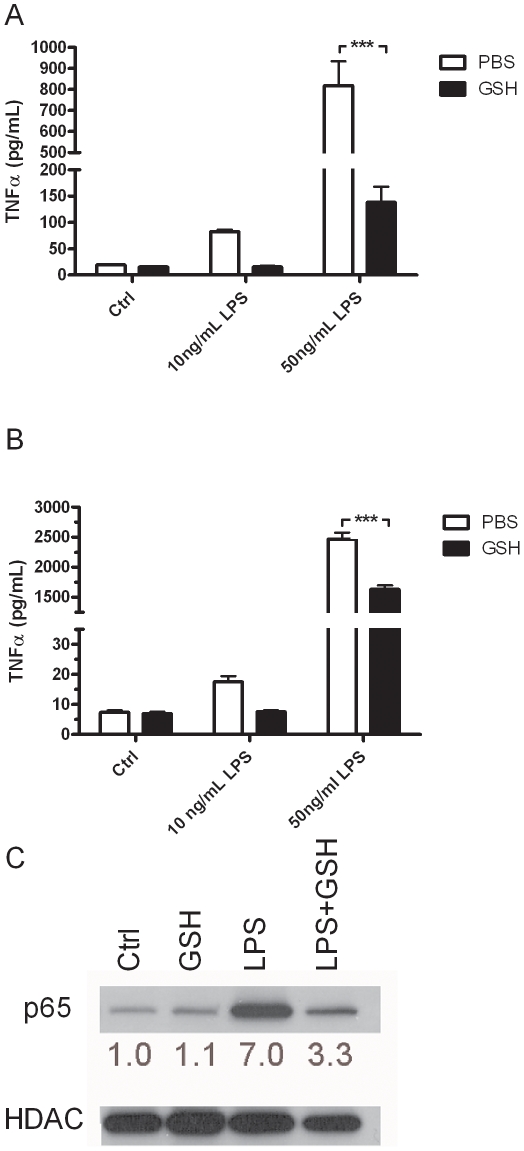
GSH supplementation alters inflammatory cytokine release. The J774 (A) and primary AM (B) were grown in media with or without 500 µM GSH and treated with LPS for 2 hours. TNFα release was analyzed in the media by ELISA. J774 nuclear fractions were examined for the nuclear translocation of the p65 subunit of NF-κB (C), HDAC used as a protein loading control with the ratio of p65 to HDAC area density listed below. Data represented as mean ± SEM, with ***p<0.001 compared between PBS and GSH.

GSH has been shown to be a potent regulator of proinflammatory cytokine release in vitro and in vivo [14], [19]. Additionally, lower GSH in the airways has previously been associated with excess macrophage activation, which can lead to proinflammatory cytokine release [14]. To confirm that GSH has a similar anti-inflammatory effect in macrophages as has been previously shown, both the J774 (Fig. 2A) and primary AM (Fig. 2B) were grown in either normal or GSH containing media and stimulated with 10 or 50 ng/mL LPS for 2 h, after which TNFα release was measured. There was a dose dependant increase in TNFα in the J774 (Fig. 2A) cells after LPS stimulation, while GSH significantly attenuated the TNFα release. Similarly, GSH also significantly attenuate the LPS mediated TNFα at both doses of LPS in the primary AM (Fig. 2B). In contrast to the cell line, the primary AM cells had a much higher TNFα response to the high dose of LPS. In both cell types the extracellular GSH supplementation blocked any increase in TNFα with 10 ng/mL LPS dose. LPS itself did not have any effect on intracellular GSH levels or uptake (data not shown). To confirm that GSH is having anti-inflammatory effects, nuclear translocation of the p65 subunit of NF-κB was examined in the J774 cells after stimulation with LPS in the presence or absence of GSH (Fig. 2C). Confirming the results of the cytokine analysis, the supplementation of GSH blocked the nuclear translocation of NF-κB.

### Intracellular GSH accumulation is dependent on macropinocytosis

**Figure 3:**
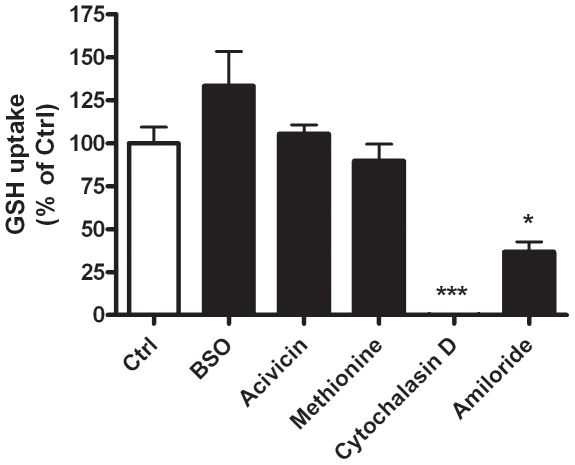
Uptake of GSH is through macropinocytosis. J774 cells were grown in media supplemented with 500 µM GSH either containing buthionine sulfoxime (BSO), acivicin, or methionine to inhibit synthesis, breakdown or transport of GSH, respectively (A). Cytochalasin D was used as an inhibitor of endocytosis and amiloride was used as more specific inhibitor of macropinocytosis. Data represented as mean ± SEM, with *p<0.05, ***p<0.001 compared to control.

To determine how macrophages can utilize the available GSH in the extracellular compartment various pathways of GSH utilization, including synthesis, breakdown, transport, and endocytosis were examined in the J774 cells (Fig. 3). Buthionine sulfoximine (BSO) is a potent inhibiter of γ-glutamylcysteine ligase, the rate limiting enzyme involved in GSH synthesis. γ-Glutamyl transferase is the only known enzyme that breaks down GSH, which can be inhibited with acivicin, and previous reports have shown that intracellular transport of GSH can be inhibited using methionine [20]. Inhibiting GSH synthesis with BSO did not alter the intracellular uptake of GSH compared to the uptake in untreated cells. Furthermore, the inhibition of either breakdown or transport did not affect the uptake of GSH. Conversely, cytochalasin D, a broad inhibitor of endocyotosis was able to completely block the uptake of GSH, suggesting that the increase in intracellular GSH is not through other more common pathways but rather through an endocytosis uptake mechanism. To further narrow down the potential endocytosis mechanism amiloride, an inhibitor of macropinocytosis, was used and shown to inhibit nearly half of the GSH uptake, strongly suggesting that the uptake mechanism is macropinocytosis. Furthermore, polyinositol acid and amantadine, two inhibitors of receptor mediated endocytosis did not block the GSH uptake (data not shown). While the most likely uptake mechanism is macropinocytosis, in comparison to amiloride, cytochalasin D is a much more potent inhibitor of GSH uptake, therefore for further inhibitory studies the more potent inhibitor was used.

### Macropinocytosis is required to attenuate inflammatory responses

**Figure 4:**
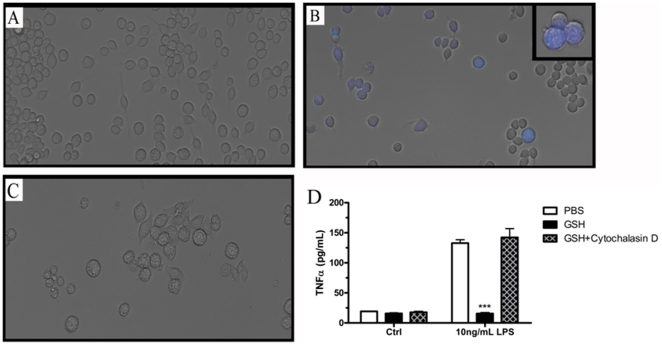
Anti-inflammatory effects of GSH are dependent on macropinocytosis. Brighfield images of control (A), fluorescent labeled GSH (Fl-GSH) (B) or Fl-GSH with cytochalasin D (C) provide further evidence for the direct uptake of extracellular GSH. Furthermore, LPS induced TNFα release was examined after supplementation with GSH and/or cytochalasin D to block that uptake (D). Data represented as mean ± SEM, with ***p<0.001 compared to either LPS only.

To further show that the GSH administered in the extracellular space is responsible for the intracellular increases, fluorescent labeled GSH was used as a probe for the macropinocytosis of GSH. Cells were incubated with a combination of unlabelled and fluorescent labeled GSH in a ratio of 4∶1 and the GSH uptake was visualized after 24 h. In contrast to the control cells (Fig. 4A) those that were incubated with GSH (Fig. 4B) showed an increase in fluorescence which could then be blocked using cytochalasin D (Fig. 4C). It is interesting to note that not all cells had increased fluorescent labeling suggesting that rates of macropinocytosis must vary among the cells.

To elucidate the role of extracellular GSH versus intracellular GSH, cytochalasin D was used to block the uptake of extracellular GSH in J774 cells and the LPS induced TNFα release was examined (Fig. 4D). The cells were grown in normal or GSH containing media with cytochalasin D to block the uptake and then stimulated with LPS for 2 h. While the cells cultured in GSH supplemented media alone sho wed an attenuation of LPS stimulated TNFα release, cytochalasin D completely blocked the anti-inflammatory effect of GSH with TNFα levels comparable to LPS stimulated cells alone, cytochalasin D alone did not have any effect on TNFα release (data not shown). Taken together these data show that the extracellular administered GSH is in fact being internalized which is required for the attenuation of cytokine release and that the anti-inflammatory effect of GSH is due entirely to the intracellular status, not the extracellular levels.

### Uptake of exogenously administered GSH *in vivo*

**Figure 5:**
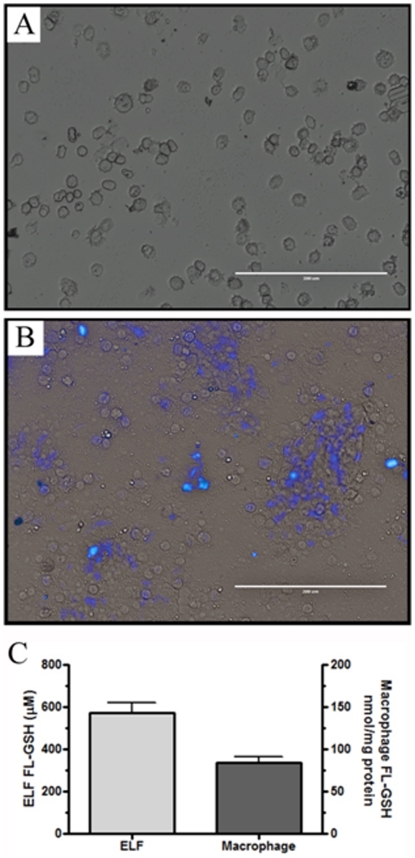
In vivo uptake of fluorescent labeled GSH. Fluorescent labeled GSH (Fl-GSH) was administered intratracheally and examined after a period of 2 h. Representative image of BAL cells recovered from control (A) and Fl-GSH (B) administered mice. Quantization of resulting Fl-GSH concentrations 2 h after administration (C), control samples contain no detectable fluorescence.

To definitively determine whether the macropinocytosis of GSH occurs in vivo, Fl-GSH was administered intratracheally and examined after a period of 2 h. Fl-GSH (20 µL) was administered to each mouse based on the assumption that the ELF is roughly 100 µL, theoretically resulting in roughly a 500 µM concentration of Fl-GSH in the ELF. The Fl-GSH was visualized using fluorescent microscopy revealing a cellular accumulation of Fl-GSH (Fig. 5B) compared with control BAL cells (Fig. 5A) which do not contain any fluorescence. The unique ability to detect the Fl-GSH over endogenous GSH was achieved by quantifying the change in fluorescence in both the ELF and BAL cells (Fig. 5C) by HPLC with fluorometric detection. The concentration of Fl-GSH in the ELF resulted in roughly 575 µM and 84 nmol/mg in the BAL cell lysates. It should be noted that this is only the amount of the exogenously administered Fl-GSH in the ELF and BAL cells, since it does not take into account the endogenous non-fluorescent GSH levels. This data shows that when exogenous GSH is administered it is readily taken up by the BAL cells which are predominately AMs.

### GSH status with two cigarette smoke exposure conditions results in altered cytokine response

**Figure 6:**
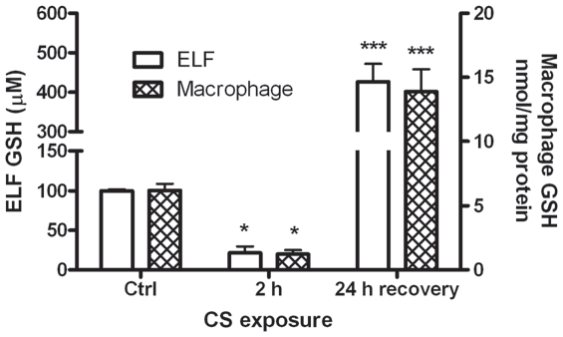
Different smoke exposure paradigms result in different GSH levels. Mice were exposed to acute 2 h cigarette smoke (CS) exposure with little rest before sampling of the ELF and BAL cell GSH which resulted in depleted GSH levels. However, after a 24 h exposure which includes a rest period there is a full adaptive response with elevated ELF and BAL cell GSH levels. Data represented as mean ± SEM, with *p<0.05, ***p<0.001 compared to either ELF or BAL cell control.

COPD is one of the most prevalent lung diseases characterized by exaggerated lung inflammation, but it also has been shown to have altered ELF GSH levels [5]. In relation to the previous data shown, AMs may be major mediators of inflammation with CS, the primary cause of COPD. Therefore, two different CS exposure paradigms were examined; an acute 2 h smoke exposure with no rest time before sampling of the ELF and a 24 h exposure with the mice exposed to CS for 5 h and allowed to rest for a complete 19 h before sampling of the ELF. With very short periods of CS, GSH can become depleted and we have established that this occurs within 2 h (data not shown); furthermore, with a slightly longer exposure it is well known the CS will induce adaptive responses, including GSH synthesis, resulting in increased ELF GSH [14]. We utilized both exposure paradigms to establish two very different ELF environment, one in which the ELF GSH is depleted down to 21 µM after the 2 h exposure and one in which the ELF GSH is increased to roughly 425 µM after a 24 h exposure (Fig. 6). As was shown in Table 1, the BAL cell GSH, which is in the intracellular compartment, also corresponds with the either increase or decrease in ELF GSH. With the 2 h exposure the BAL cell GSH falls to roughly 1.2 nmol/mg while the BAL cell GSH is increased to roughly 11.5 nmol/mg after the full 24 h exposure.

**Figure 7:**
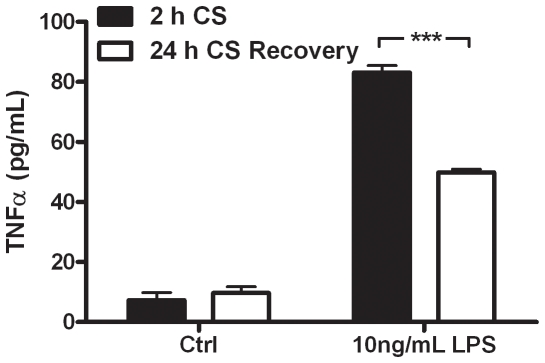
Altered GSH status in the airways results in different cytokine response to LPS. Alveolar macrophages were obtained from mice sampled immediately after a 2 h (closed bars) cigarette smoke (CS) exposure or 24 h (open bars) recovery from CS exposure and treated with LPS for 2 h. TNFα release was measured by ELISA. Data represented as mean ± SEM, with ***p<0.001 compared between 2 h CS and 1 d CS.

These two CS exposure paradigms were utilized to determine whether altered extracellular GSH levels have an effect on macrophage cytokine production in an ex vivo model. Primary AM were isolated from mice either exposed to CS for 2 or 24 h and incubated with LPS in culture media for 2 h, after which TNFα release was examined (Fig. 7). The AM that were in the low GSH ELF after the 2 h exposure had a higher TNFα response to LPS than the cells that had resided in the high ELF GSH after the 24 h exposure. Neither of the cell conditions had altered basal levels of TNFα release. This data shows that the utilization of GSH to modulate inflammatory cytokine release can occur in vivo and may have a significant impact on inflammatory lung diseases that have characteristically low ELF GSH levels.

## Discussion

These studies demonstrate a strong association between the extracellular (ELF) and intracellular GSH levels in AMs. In addition, these studies provide data that suggests macrophages can utilize extracellular GSH to supplement intracellular GSH levels via macropinocytosis that redox modulates NF-kB signaling involved in inflammatory responses to triggers such as LPS. This evidence supports a novel mechanism by which extracellular GSH status modulates AM response to inflammatory stimuli and provides a role for extracellular GSH as a negative feedback loop for lung inflammation.

The GSH status of the ELF has recently been shown to correlate with the amount of TNFα secreted in the airways [14]. Additionally, there is an increase in activation state of the AM from mice with low ELF GSH in response to CS compared to those from mice with higher ELF GSH. In the present study, the maintenance of macrophage intracellular GSH levels seems to be partly dependent on the ELF GSH pool which the AM can access by endocytosis. The utilization of extracellular GSH by the AM is interesting since they posses all the necessary enzymes and cofactors for de novo synthesis of GSH. One possibility is that in contrast to some other apical fluids, like plasma, the ELF contains rather low levels of cysteine/cystine [21], thus while the AM do have the ability to synthesize GSH, the constitutive amino acids may be limiting. There is also some evidence that inflammatory cells may not express the enzymes needed for GSH synthesis to the same degree as other cell types, and low enzyme expression limits the de novo synthesis ability [22], [23]. The lung may use this auxotrophy as a means to regulate inflammatory responses to environmental stimuli.

Macrophages make up the majority of the cell types found in the alveolar spaces under normal conditions. Because of the large population of macrophages compared to other leukocytes like neutrophils, the macrophages are most likely a major mediator of inflammatory signaling in the lung [15], [24], [25], [26]. While previous reports have shown that thiol status can alter cytokine release [27], this is the first report to link it with a potential in vivo consequence. Macrophages are well known scavengers of particles, viruses, and bacteria within the lung, thus they have specialized mechanisms such as phagocytosis to achieve these functions. There are many different classes of endocytosis including receptor mediated, receptor independent, pinocytosis, and macropinocytosis just to name a few [28]. Due to the abundance of GSH in the ELF, it does not seem conceivable that receptor mediated pathways would be necessary for the uptake of GSH, therefore the uptake is most likely a generalized sampling of the extracellular fluid. In the present study, the uptake of GSH is blocked by cytochalasin D, an inhibitor of actin polymerization, indicating that pinocytosis can be ruled out since actin is not required for the uptake of small particles [28]. However, an uptake mechanism of slightly larger particles up to 2 µm in size, which does require actin is macropinocytosis [29]. Since GSH was observed to be taken up, and it is well under the 2 µm size of vesicles that form, it stands to reason that the mechanism of endocytosis is macropinocytosis. Amiloride has been reported to be an inhibitor of macropinocytosis [30], however it did not completely block GSH uptake. Amiloride works primarily by blocking sodium channels and altering intracellular pH affecting the activity of certain kinases involved so it is conceivable that other effects not related to the inhibition of macropinocytosis could be involved. Regardless, cytochalasin D did completely inhibit uptake which strongly suggests the uptake mechanism is macropinocytosis.

The ability for cytochalasin D to block the GSH mediated attenuation of cytokine release shows that it is the intracellular thiol status of the macrophages that is important for the modulation of inflammation. Any of the extracellular effects of GSH, such as the potential to alter receptors or binding to the membrane can be ruled out since blocking the uptake of GSH with cytochalasin D resulted in a similar TNFα level as LPS alone. Furthermore, this also shows that GSH does not affect LPS or surface receptors on the extracellular membrane. Increases in cellular thiol status have previously been shown to block NF-κB, MAP kinase, and phosphatase activity as well, also pointing to an intracellular effect rather than an extracellular one [31], [32], [33], [34], [35]. While the mechanism by which thiol status attenuates inflammation is not exactly clear, several theories have been proposed. There is some suggestion that thiols protect redox sensitive phosphatases or modulate kinase activation, resulting in modulated NF-κB activity [36]. There is also some evidence that GSH may be detoxifying hydrogen peroxide and other ROS produced from membrane associated NADPH oxidase enzymes, thus preventing the downstream signaling events that lead to activation of certain proinflammatory pathways [37], [38]. While the detoxification of ROS would certainly fit with the antioxidant properties of GSH, not all inflammatory stimuli have a large ROS component. For instance, certain activators like PMA have been shown to cause an increase in ROS [20], while others such as LPS used in the current study are not typically associated with large increases in ROS [39]. Additionally, in the current study, LPS did not have any effect on intracellular levels of GSH, which would not be expected if there was a large ROS component to the activation. Therefore, while the intracellular GSH status is the critical component to the modulation of inflammation, it is still not exactly clear how GSH prevents the activation of NF-κB.

The fact that the intracellular GSH status is important for the modulation of GSH provides an interesting dynamic, especially in the lung. It is well known the ELF GSH is maintained at much higher levels than the corresponding plasma GSH [5], but there has really been very rationale little to explain this. Previously, GSH was seen mainly as a defense mechanism against the high oxygen tension and potential oxidative stress from inhaled gases, particulates, and pathogens in the lung. The current study would suggest an inter-relationship between the epithelial cells, which supply the bulk of the ELF GSH, and the resident AMs that can utilize that GSH. The reliance of the macrophages on the GSH that is supplied by the epithelial cells indicates another role for GSH in the ELF. For instance, modulating ELF GSH levels could lead to modulating the response to certain inflammatory stimuli. While inflammatory signaling is an important part of the immune system in many lung diseases this signaling system becomes dysregulated and prolonged which contributes to the progression of lung diseases [24], [40]. The triggers that either prevent or exacerbate inflammatory signals are numerous and the current studies suggest GSH status of the airway may be an important aspect in its regulation.

There are numerous lung diseases with dysregulated ELF GSH, such as cystic fibrosis (CF), acute respiratory distress syndrome (ARDS), idiopathic pulmonary fibrosis (IPF), or aging [5]. Many of these diseases are also associated with increased or exaggerated inflammatory responses and one could postulate that it may be the altered macrophage GSH status that could be contributing to the dysregulated inflammation. One of the more interesting aspects of the potential contribution to diseases might be with CF. With CF mutations the apical transport of GSH is altered, which decreases ELF GSH levels, but in theory that should not affect the intracellular levels of macrophages, and yet the macrophage GSH levels are decreased as much as all of the other models examined. Furthermore, macrophages have been implicated in promoting the progression of CF [26], [41], [42]. This could suggest that finding ways to alter ELF GSH levels in inflammatory lung diseases like CF or COPD, one could potentially regulate inflammatory responses with the goal to slow or prevent the progression of inflammatory lung diseases. Although a few clinical trials of inhaled GSH have been done in CF subjects, the results have been only modestly positive [43], [44]. A potential problem with inhalation delivery of GSH is the large amount of oxidation that is reported to occur which may limit the amount of the active reduced form of GSH in the ELF [45]. New therapeutic approaches that stimulate lung epithelial cells to secrete the active reduced form of GSH directly into the ELF will be needed to overcome this obstacle. We have recently reported that hypertonic saline nebulization increases ELF GSH by this type of mechanism [46]. These types of therapeutic approaches may provide effective therapeutic options for a whole host of lung disease associated with low ELF GSH levels including COPD, lung fibrosis, ARDS and CF.
